# Differential roles of RIPK1 and RIPK3 in TNF-induced necroptosis and chemotherapeutic agent-induced cell death

**DOI:** 10.1038/cddis.2015.16

**Published:** 2015-02-12

**Authors:** K Moriwaki, J Bertin, P J Gough, G M Orlowski, F KM Chan

**Affiliations:** 1 Department of Pathology, Immunology and Microbiology Program, University of Massachusetts Medical School (UMMS), Worcester, MA 01655, USA; 2 Pattern Recognition Receptor Discovery Performance Unit, Immuno-Inflammation Therapeutic Area, GlaxoSmithKline, Collegeville, PA 19422, USA

## Abstract

Apoptosis is a key mechanism for metazoans to eliminate unwanted cells. Resistance to apoptosis is a hallmark of many cancer cells and a major roadblock to traditional chemotherapy. Recent evidence indicates that inhibition of caspase-dependent apoptosis sensitizes many cancer cells to a form of non-apoptotic cell death termed necroptosis. This has led to widespread interest in exploring necroptosis as an alternative strategy for anti-cancer therapy. Here we show that in human colon cancer tissues, the expression of the essential necroptosis adaptors receptor interacting protein kinase (RIPK)1 and RIPK3 is significantly decreased compared with adjacent normal colon tissues. The expression of RIPK1 and RIPK3 was suppressed by hypoxia, but not by epigenetic DNA modification. To explore the role of necroptosis in chemotherapy-induced cell death, we used inhibitors of RIPK1 or RIPK3 kinase activity, and modulated their expression in colon cancer cell lines using short hairpin RNAs. We found that RIPK1 and RIPK3 were largely dispensable for classical chemotherapy-induced cell death. Caspase inhibitor and/or second mitochondria-derived activator of caspase mimetic, which sensitize cells to RIPK1- and RIPK3-dependent necroptosis downstream of tumor necrosis factor receptor-like death receptors, also did not alter the response of cancer cells to chemotherapeutic agents. In contrast to the RIPKs, we found that cathepsins are partially responsible for doxorubicin or etoposide-induced cell death. Taken together, these results indicate that traditional chemotherapeutic agents are not efficient inducers of necroptosis and that more potent pathway-specific drugs are required to fully harness the power of necroptosis in anti-cancer therapy.

Cell death by apoptosis is a natural barrier to cancer development, as it limits uncontrolled proliferation driven by oncogenes.^1^ Chemotherapeutic agents that target apoptosis have been successful in anti-cancer therapy. However, cancer cells, especially cancer stem cells, often evolve multiple mechanisms to circumvent growth suppression by apoptosis.^2^ This resistance to apoptosis is a major challenge for many chemotherapeutic agents. Targeting other non-apoptotic cell death pathways is an attractive therapeutic alternative.

A growing number of recent studies show that there are distinct genetic programmed cell death modes other than apoptosis.^3^ Necroptosis is mediated by receptor interacting protein kinase 3 (RIPK3).^4^ In the presence of caspase inhibition and cellular inhibitor of apoptosis proteins (cIAPs) depletion, tumor necrosis factor (TNF) receptor 1 triggers a signaling reaction that culminates in binding of RIPK3 with its upstream activator RIPK1 through the RIP homotypic interaction motif (RHIM).^4^ RIPK1 and RIPK3 phosphorylation stabilizes this complex and promotes its conversion to an amyloid-like filamentous structure termed the necrosome.^5^ Once activated, RIPK3 recruits its substrate mixed lineage kinase domain-like (MLKL).^6^ Phosphorylated MLKL forms oligomers that translocate to intracellular membranes and the plasma membrane, which eventually leads to membrane rupture.^7, 8, 9, 10^

In addition to phosphorylation, RIPK1 and RIPK3 are also tightly regulated by ubiquitination, a process mediated by the E3 ligases cIAP1, cIAP2, and the linear ubiquitin chain assembly complex.^11^ The ubiquitin chains on RIPK1 act as a scaffold to activate nuclear factor-*κ*B (NF-*κ*B) and mitogen-activated protein kinase pathways and inhibit formation of the necrosome. As such, depletion of cIAP1/2 by second mitochondria-derived activator of caspase (Smac) mimetics or removal of the ubiquitin chains by the de-ubiquitinating enzyme cylindromatosis (CYLD) promotes necroptosis.^12, 13, 14, 15^ In addition, RIPK1 and RIPK3 are cleaved and inactivated by caspase 8.^16, 17, 18^ Mice deficient for caspase 8 or FADD, an essential adaptor protein of caspase 8, suffer from embryonic lethality due to extensive RIPK1- or RIPK3-dependent necroptosis.^19, 20, 21^ Hence, caspase inhibition and IAP depletion are key priming signals for necroptosis.

The physiological functions of RIPK1 and RIPK3 have been extensively investigated in infectious and sterile inflammatory diseases.^4, 22^ By contrast, their roles in cancer cells' response to chemotherapeutics are poorly understood. Here we show that RIPK1 and RIPK3 expression is significantly decreased in human colon cancer tissues, suggesting that suppression of RIPK1 or RIPK3 expression is advantageous for cancer growth. However, the loss of RIPK1 and RIPK3 expression in colon cancer was not due to epigenetic DNA modification. Interestingly, RIPK1 and RIPK3 expression in colon cancer cells is reduced by hypoxia, a hallmark of solid tumor. We found that chemotherapeutic agents did not effectively elicit RIPK1/RIPK3-dependent necroptosis in colon cancer cells. Moreover, caspase inhibition and Smac mimetics, which are potent sensitizers for necroptosis, also did not enhance chemotherapeutic agent-induced cell death. These results show that traditional chemotherapeutic agents are not strong inducers of classical necroptosis in colon cancers and suggest that development of pathway-specific drugs is needed to harness the power of necroptosis in anti-cancer therapy.

## Results

### Loss of RIPK1 and RIPK3 expression in human colon cancer tissues

**Figure 1:**
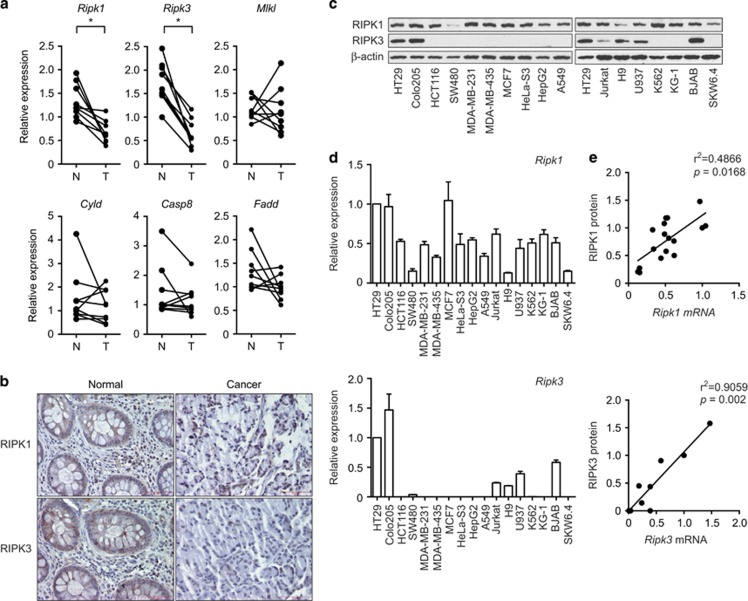
The expression of *Ripk1* and *Ripk3* is decreased in human colon cancer. (**a**) Total RNA from human colon cancer tissues (T) and adjacent normal colon tissues (N) were analyzed by real-time PCR for the expression of *Ripk1*, *Ripk3*, *Mlkl*, *Cyld*, *Casp8*, and *Fadd*. *P-*values were calculated by Wilcoxon matched-pairs signed-rank test. *P<0.05. (**b**) Pictures of normal and cancer tissues stained for RIPK1 and RIPK3 are shown. Bars: 75 *μ*M. (**c**) Whole-cell extracts and (**d**) RNA from various cancer cell lines were analyzed by western blotting and real-time PCR, respectively, for the expression of RIPK1 and RIPK3. For comparison between different cell lines, we define the expression in HT29 cells as 1. (**e**) Comparison of mRNA and protein level of RIPK1 (upper panel) and RIPK3 (lower panel). The intensity of RIPK1 and RIPK3 protein expression in **c** was quantified using ImageJ. The protein expression level in HT29 cells was defined as 1. *P*-value and *r*^2^ were determined by Spearman correlation coefficients. Error bars indicate S.E.M. (*n*=3)

To investigate the role of necroptosis in colon cancer, we examined the mRNA expression level of central regulators of necroptosis in human colon cancer tissues and paired adjacent normal colon tissues by real-time PCR (Supplementary Table S1). We found that mRNA expression of *Ripk1* and *Ripk3* was significantly decreased in colon cancer tissues compared with paired normal colon tissues (*P*=0.0039 for *Ripk1* and *Ripk3* by Wilcoxon matched-pairs signed-rank test; Figure 1a). In contrast, no significant differences were observed for the expression of *Mlkl* (*P*=0.7344), *Cyld* (*P*=0.4258), *Casp8* (*P*=0.3008), and *Fadd* (*P*=0.1641) (Figure 1a). Immunohistochemical analysis confirmed that RIPK1 and RIPK3 were expressed in colonic enterocytes and lamina propria mononuclear cells in normal tissue, and that their expression was reduced in cancer tissues (Figure 1b). Protein expression is controlled by transcriptional and posttranscriptional mechanisms. We compared RIPK1 and RIPK3 protein, and mRNA expression in 17 different epithelial cancer and lymphoma cell lines and found that RIPK1 mRNA and protein were ubiquitously detected in all cancer cell lines tested (Figures 1c and d). In contrast, RIPK3 mRNA and protein expression was limited to 2 out of 10 epithelial cancer cell lines (HT29 and Colo205) and 4 out of 7 lymphoma cell lines (Jurkat, H9, U937, and BJAB). Although there are some exceptions, both *Ripk1* and *Ripk3* mRNA expression was well correlated with their protein expression across different tumor lines (*P*=0.0168 for RIPK1 and *P*=0.002 for RIPK3 by Spearman correlation coefficients; Figure 1e). These results suggest that RIPK1 and RIPK3 protein expression in tumor cells is mainly controlled by transcription.

### RIPK1 and RIPK3 expression is inhibited by hypoxia

**Figure 2:**
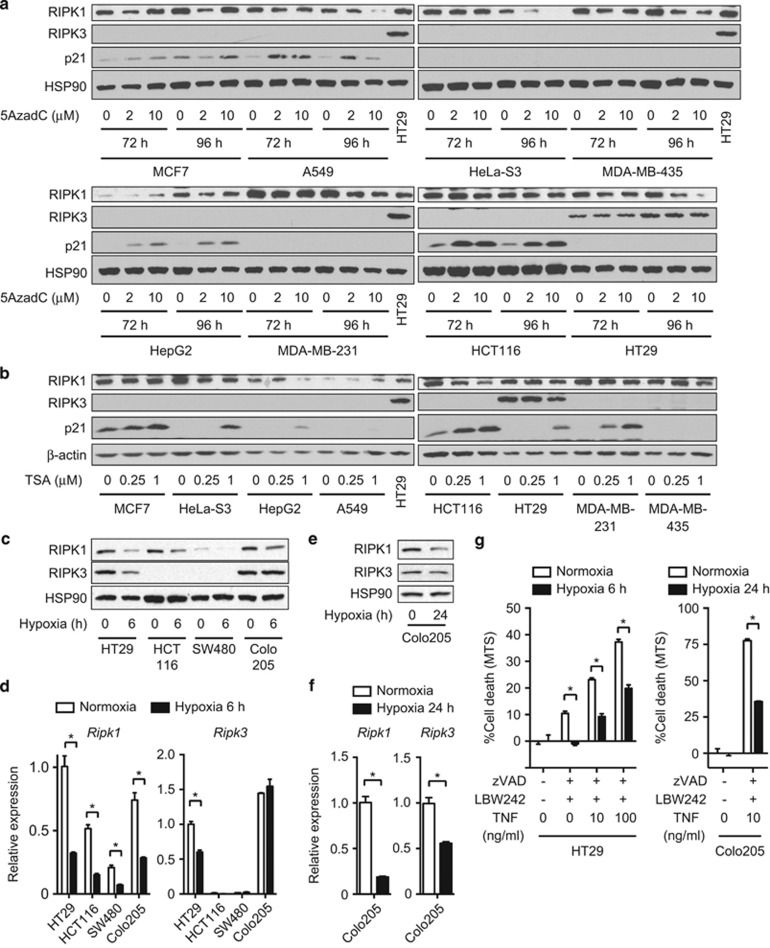
RIPK1 and RIPK3 expression is regulated by hypoxia, but not by DNA methylation or histone deacetylation. (**a**) The cancer cell lines were treated with 5AzadC as indicated. (**b**) The cells were treated with TSA for 24 h. RIPK1, RIPK3, and p21 expression was determined by western blotting. (**c–f**) Cells were exposed to 1% O_2_ hypoxic condition for (**c** and **d**) 6 or (**e** and **f**) 24 h. (**c** and **e**) Whole-cell extracts and (**d** and **f**) RNA were prepared for western blotting and Q-PCR, respectively. (**g**) Necroptosis was induced by pretreatment with 20 *μ*M zVAD and 10 *μ*M LBW242 for 1 h, followed by treatment with TNF overnight. For hypoxia, HT29 cells and Colo205 cells were cultured in 1% O_2_ hypoxic condition for 6 and 24 h before and during necroptosis induction, respectively. Cell death was determined using CellTiter96 Aqueous Non-Radioactive Cell Proliferation Assay. Error bars indicate S.E.M. (*n*=3). *P<0.05

Expression of tumor suppressor genes is often silenced in cancer tissues by epigenetic DNA modifications such as DNA methylation and histone deacetylation.^23^ In fact, an early report suggests that the promoter of *Ripk3* is hypermethylated,^24^ suggesting that RIPK1 and RIPK3 expression is epigenetically regulated. However, the DNA methylation inhibitor 5-Aza-2′-deoxycytidine (5AzadC) and histone deacetylase inhibitor trichostatin A (TSA) did not restore RIPK1 and RIPK3 expression in multiple tumor cell lines (Figures 2a and b). Consistent with previous reports,^25, 26^ 5AzadC and TSA strongly induced the expression of the cyclin-dependent kinase inhibitor p21 in many cell types (Figures 2a and b). These results indicate that the loss of RIPK1 and RIPK3 expression in colon cancer cells is not due to epigenetic DNA modifications.

Poor vascularization leads to severe oxygen deprivation or hypoxia, and is a stress confronted by solid tumors. Hypoxia has been implicated to promote cancer progression by activating adaptive transcriptional programs that promote cell survival and angiogenesis.^27^ Indeed, we found that exposure to hypoxia (1% O_2_) for 6 h reduced the expression of RIPK1 in the colon carcinoma cell lines HT29, HCT116, SW480, and Colo205 (Figure 2c). RIPK3 expression was also suppressed in HT29 cells (Figure 2c). Real-time PCR analysis revealed that diminished RIPK1 and RIPK3 protein expression was due to reduced transcription (Figure 2d). In Colo205 cells, RIPK3 mRNA and protein expression was not decreased 6 h after hypoxia exposure. Prolonged exposure to hypoxia for 24 h significantly decreased *Ripk3* mRNA expression, although protein expression was minimally affected (Figures 2e and f). The reduction in RIPK1 and RIPK3 expression was functionally significant, because necroptosis induced by TNF, the pan caspase inhibitor z-VAD-fmk (zVAD), and the Smac mimetic LWB242 was suppressed under hypoxic condition (Figure 2g). Hence, *Ripk1* and, to a lesser extent, *Ripk3* expression is regulated by hypoxia.

### RIPK activities are dispensable for chemotherapeutic agent-induced cell death

**Figure 3:**
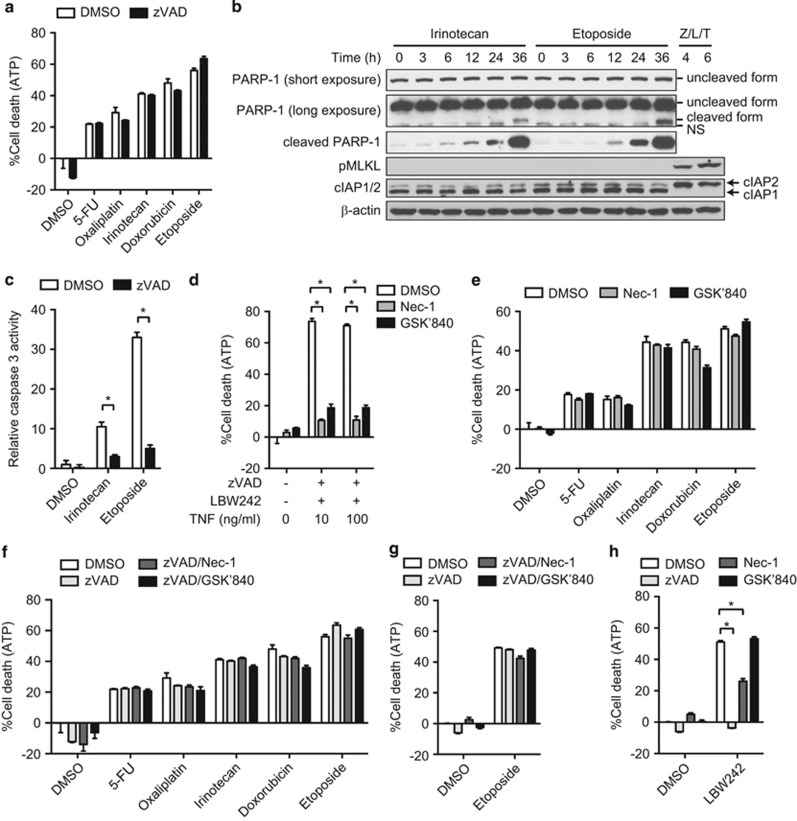
Chemotherapeutic agents induce caspase-independent non-necroptotic cell death. (**a**) HT29 cells were pretreated with zVAD before treatment with various chemotherapeutic agents. (**b**) Whole-cell extracts were prepared from HT29 cells treated with irinotecan or etoposide, and analyzed by western blotting. Necroptosis was induced by zVAD, LBW242, and TNF (Z/L/T). (**c**) Caspase 3 activity was quantified as described in Materials and Methods, in HT29 cells treated with irinotecan or etoposide for 36 h in the absence or the presence of zVAD. (**d** and **e**) Cell death was induced by (**d**) zVAD, LBW242, and TNF, or (**e**) the indicated chemotherapeutic agents in the absence or presence of Nec-1 or GSK'840 in HT29 cells. (**f**) HT29 and (**g**) MDA-MB-231 cells were pretreated with zVAD in combination with Nec-1 or GSK'840 before treatment with the chemotherapeutic agents. (**h**) MDA-MB-231 cells were pretreated with zVAD in combination with Nec-1 or GSK'840, and subsequently treated with 2.5 *μ*M LBW242 for 48 h. Cell death was determined using CellTiter-Glo Luminescent Cell Viability Assay in **a** and **d–h**. Error bars indicate S.E.M. (*n*=3). *P<0.05

Recent evidence suggests that classical chemotherapeutic agents induce not only apoptosis but also non-apoptotic death.^28^ Consistent with this notion, we found that in HT29 cells zVAD did not block cell death induced by multiple chemotherapeutic agents, including the nucleoside analog 5-fluorouracil (5-FU), platinum-based agent oxaliplatin, topoisomerase I inhibitor irinotecan, anthracycline antibiotic doxorubicin, and topoisomerase II inhibitor etoposide (Figure 3a and Supplementary Figures S1a and b). This is despite clear signs of caspase-mediated cleavage of poly (ADP-ribose) polymerase-1 (PARP-1) and caspase 3 activity (Figures 3b and c). In fact, cell death was not blocked by as much as 100 *μ*M of zVAD (Supplementary Figure S1c). As necroptosis is optimally induced when the apoptotic machinery is compromised,^4^ we assessed the contribution of necroptosis to chemotherapeutic agent-induced cell death. Consistent with the essential role of RIPK1 and RIPK3 kinase activities in necroptosis, the RIPK1 kinase inhibitor necrostatin-1 (Nec-1) and RIPK3 kinase inhibitor GSK'840 (Mandal *et al.*^29^) efficiently blocked TNF-, zVAD-, and LBW242-induced necroptosis in HT29 cells (Figure 3d). In contrast, chemotherapeutic agent-induced cell death was not inhibited by Nec-1 or GSK'840 (Figure 3e and Supplementary Figure S1d). In addition, unlike TNF, zVAD, and LBW242, neither irinotecan nor etoposide induced phosphorylation of MLKL, a signature of necroptosis (Figure 3b). Moreover, Nec-1 and GSK'840 also did not protect HT29 cells from chemotherapeutic agent-induced cell death in the presence of zVAD (Figure 3f and Supplementary Figure S1d). These data indicate that zVAD did not switch chemotherapeutic agent-induced cell death from apoptosis to necroptosis.

Recently, certain chemotherapeutic agents such as etoposide were shown to promote assembly of a macromolecular complex termed the ripoptosome in some cell lines, including MDA-MB-231. Chemotherapeutic agent-induced assembly of the ripoptosome involves degradation of cellular IAP proteins and RIPK1 kinase activity. Depending on the activity of caspase 8, the ripoptosome can drive cells towards apoptosis or necroptosis.^30^ Surprisingly, cIAP1 and cIAP2 expression was not affected by etoposide in HT29 cells (Figure 3b). By contrast, LBW242 caused degradation of cIAP1, but not NF-*κ*B-dependent re-expression of cIAP2 (Figure 3b), as cIAP1 is required for cIAP2 degradation.^31^ In addition, we found that zVAD alone or in combination with Nec-1 or GSK'840 did not protect MDA-MB-231 cells against etoposide-induced cell death (Figure 3g). Smac mimetic induces RIPK1, but not RIPK3, kinase activity-dependent apoptosis through autocrine TNF production in MDA-MB-231 cells.^32, 33^ In agreement with these observations, LBW242-induced cell death was suppressed by Nec-1, but not GSK'840 (Figure 3h). These results indicate that neither caspases nor the RIP kinases are required for chemotherapeutic agent-induced cell death in colon cancer cells.

### Smac mimetic enhances irinotecan-induced cell death in a RIP kinase-independent manner

**Figure 4:**
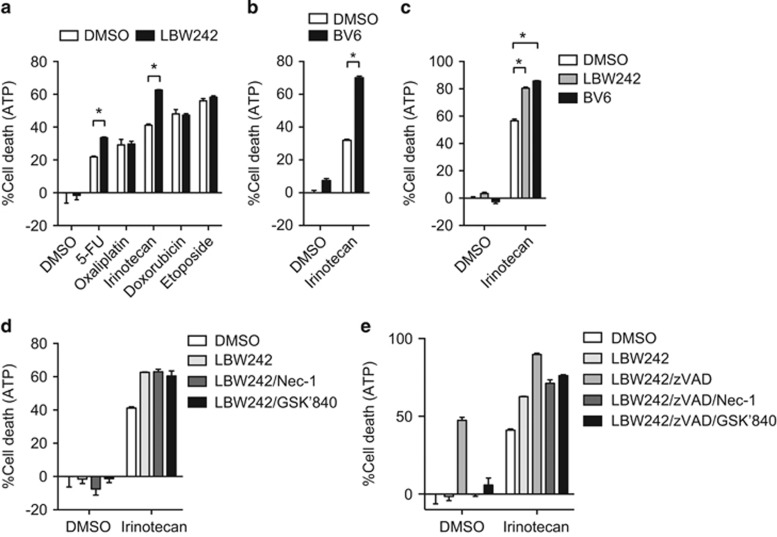
Smac mimetics enhance cancer cell death in a chemotherapeutic agent-specific manner. (**a** and **b**) HT29 cells or (**c**) Colo205 cells were pretreated with (**a** and **c**) 2.5 *μ*M LBW242 or (**b** and **c**) 0.2 *μ*M BV6 for 1 h and subsequently treated with chemotherapeutic agents. (**d**) HT29 cells pretreated with 2.5 *μ*M LBW242 in the presence of Nec-1 or GSK'840 were treated with chemotherapeutic agents. (**e**) Cells were treated as in **d**, except that zVAD was added where indicated. Cell death was determined using CellTiter-Glo Luminescent Cell Viability Assay. Error bars indicate S.E.M. (*n*=3). *P<0.05

Smac mimetics trigger auto-ubiquitination and degradation of cellular IAPs and can greatly sensitize cancer cells to apoptosis or necroptosis.^34^ We therefore asked whether Smac mimetics could sensitize colon cancer cells to chemotherapeutic agent-induced cell death. We found that LBW242 increased cell death induced by irinotecan and, to a lesser extent, 5-FU in HT29 cells (Figure 4a). Similar increase in irinotecan-induced cell death was observed with another Smac mimetic BV6 (Figure 4b) and in another RIPK3-positive colon cancer cell line Colo205 (Figure 4c). By contrast, Smac mimetics did not enhance cell death induced by oxaliplatin, doxorubicin, or etoposide (Figure 4a). RIPK1 kinase activity was reported to be required for Smac mimetics and anticancer drug-induced apoptosis in acute lymphoblastic leukemia cells.^35^ In contrast to this report, neither Nec-1 nor GSK'840 protected Smac mimetic and irinotecan-induced cell death (Figure 4d). Addition of zVAD to either Nec-1 or GSK'840 also did not inhibit Smac mimetic and irinotecan-induced cell death (Figure 4e). Hence, Smac mimetic sensitized colon cancer cells to irinotecan-induced cell death in a caspase and RIPK-independent manner.

### RIPK1 and RIPK3 are dispensable for chemotherapeutic agent-induced cell death

**Figure 5:**
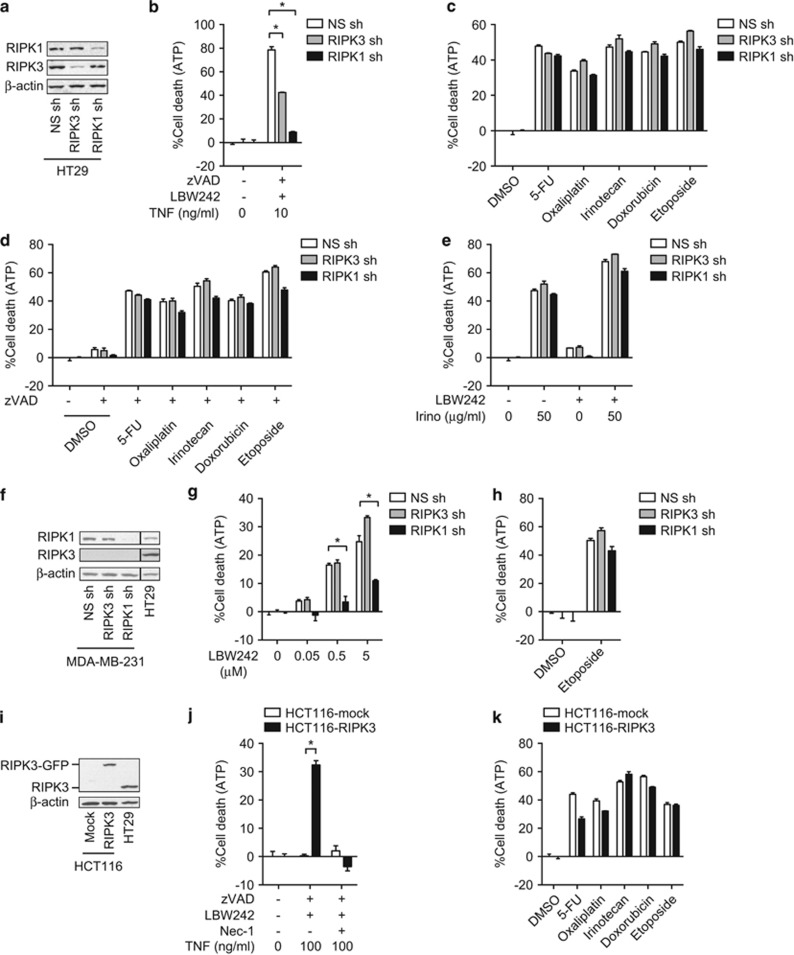
RIPK1 and RIPK3 are dispensable for chemotherapeutic agent-induced cell death. (**a**) Knockdown of RIPK1 or RIPK3 by shRNA in HT29 cells was confirmed by western blotting. (**b**) Necroptosis was induced by TNF, zVAD, and LBW242. (**c** and **d**) RIPK1 or RIPK3-knockdown HT29 cells were treated with the chemotherapeutic agents in the (**c**) absence or (**d**) presence of zVAD. (**e**) RIPK1 or RIPK3-knockdown HT29 cells were pretreated with 2.5 *μ*M LBW242 for 1 h before irinotecan treatment. (**f**) RIPK1 and RIPK3 expression in MDA-MB-231 cells stably transfected with shRNA against RIPK1 or RIPK3. The lanes were run on the same gel but were non-contiguous. (**g** and **h**) RIPK1 or RIPK3-knockdown MDA-MB-231 cells were treated with (**g**) LBW242 or (**h**) etoposide for 48 h. (**i**) Overexpression of RIPK3-GFP in HCT116 cells was confirmed by western blotting. (**j**) Necroptosis was induced by TNF, zVAD-fmk, and LBW242 in RIPK3-GFP-expressing HCT116 cells. (**k**) RIPK3-GFP-expressing HCT116 cells were treated with the indicated chemotherapeutic agents. Cell death was determined using CellTiter-Glo Luminescent Cell Viability Assay. Error bars indicate S.E.M. (*n*=3). *P<0.05

Although RIPK1 and RIPK3 kinase inhibitors had no effects on chemotherapeutic agent-induced cell death, recent evidence suggests that both RIPK1 and RIPK3 could signal independent of their kinase activities.^29, 36, 37^ To address whether kinase-independent signaling by RIPK1 and RIPK3 might contribute to chemotherapeutic agent-induced cell death, we used short hairpin RNA (shRNA) to stably silence RIPK1 or RIPK3 expression in HT29 cells (Figure 5a). Knockdown of RIPK1 or RIPK3 inhibited TNF-, zVAD-, and LBW242-induced necroptosis (Figure 5b), but had no effect on chemotherapeutic agent-induced cell death, regardless of whether zVAD was used (Figures 5c and d). RIPK1 or RIPK3 knockdown also did not inhibit irinotecan and Smac mimetic-induced cell death in HT29 cells (Figure 5e). Although Smac mimetic-induced cell death in MDA-MB-231 cells was efficiently inhibited by RIPK1 knockdown (Figures 5f and g), etoposide-induced cell death was not (Figure 5h). This is in contrast to an early report that RIPK1 or RIPK3 shRNA blocked etoposide-induced cell death in MDA-MB-231 cells.^30^ The discrepant results are likely due to differential RIPK3 expression in the MDA-MB-231 cells used in the two studies (Figures 1b and 5f).^38^

To further address the role of RIPK3 in chemotherapeutic agent-induced cell death, we stably expressed RIPK3 in the RIPK3-negative cancer cell lines HCT116, SW480, and MDA-MB-231 (Figure 5i and Supplementary Figure S2a). We found that RIPK3 reconstitution sensitized these cells to TNF-, zVAD-, and LBW242-induced necroptosis (Figure 5j and Supplementary Figure S2b). By contrast, it did not enhance chemotherapeutic agent-induced cell death (Figure 5k and Supplementary Figure S2c). These results indicate that RIPK1 and RIPK3 do not have major roles in chemotherapeutic agent-induced cell death in colon cancer cells.

### Cathepsins contribute to chemotherapeutic agent-induced cell death

**Figure 6:**
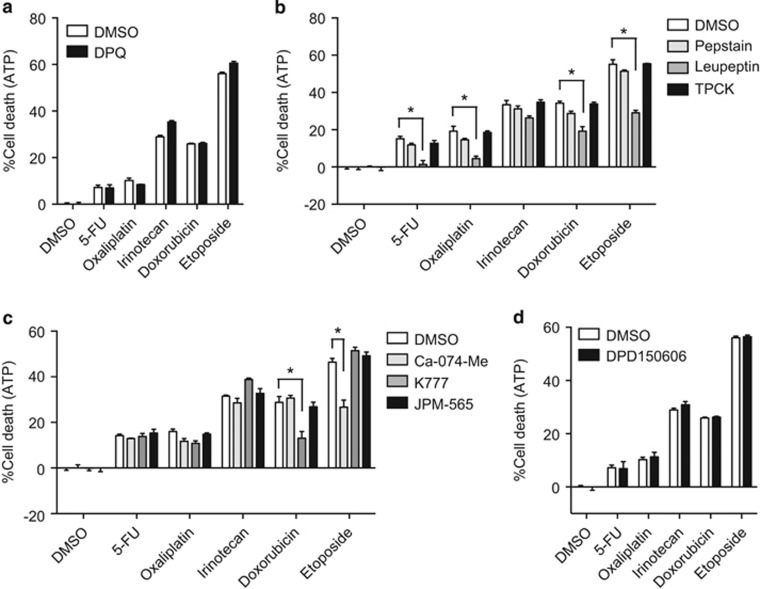
Doxorubicin and etoposide induced cathepsin-dependent cell death. HT29 cells were pretreated with (**a**) 10 *μ*M DPQ, (**b**) 1 *μ*M pepstatin, 10 *μ*M leupeptin, or 10 *μ*M TPCK, (**c**) 10 *μ*M Ca-074-Me, 10 *μ*M K777, or 10 *μ*M JPM-565, or (**d**) 10 *μ*M PD150606 for 1 h before treatment with the chemotherapeutic agents. Cell death was determined using CellTiter-Glo Luminescent Cell Viability Assay. Error bars indicate S.E.M. (*n*=3). *P<0.05

Hyperactivation of PARP-1 results in caspase-independent non-necroptotic cell death, which involves depletion of NAD^+^ and cellular ATP.^39^ However, the PARP-1 inhibitor DPQ did not suppress chemotherapeutic agent-induced cell death (Figure 6a). As non-caspase proteases have been shown to initiate or propagate cell death signals,^40^ we asked whether other proteases might be responsible for cell death induced by chemotherapeutic agents. We found that the cysteine protease inhibitor leupeptin significantly inhibited cell death induced by 5-FU, oxaliplatin, doxorubicin, and etoposide, and, to a lesser extent, irinotecan (Figure 6b). In contrast, the aspartate protease inhibitor pepstatin and the serine protease inhibitor TPCK did not affect chemotherapy-induced cell death (Figure 6b). Cathepsins and calpains are cysteine proteases that have been implicated in caspase-independent cell death.^40^ We found that the cysteine cathepsin inhibitors Ca-074-Me and K777, but not the cell-impermeable cathepsin inhibitor JPM-565 or the calpain inhibitor PD150606, partially blocked cell death induced by etoposide and doxorubicin, respectively (Figures 6c and d). Hence, in colon cancer cells, the chemotherapeutic agents tested predominantly elicit caspase- and RIPK-independent cell death that is partially dependent on cathepsins.

## Discussion

In this study, we found that RIPK1 and RIPK3 expression was reduced in primary colon cancer tissues compared with normal adjacent tissues. Similar reduction in RIPK3 expression was also reported in patients with acute myeloid leukemia.^41^ These observations are consistent with the fact that the majority of cancer cell lines lack RIPK3 expression. Intriguingly, amino acid changing mutations for RIPK3 and RIPK1 have been found in human cancer tissues according to the COSMIC database (http://cancer.sanger.ac.uk/cancergenome/projects/cosmic/). One of these mutations for RIPK3, V458M, resides within the tetra-peptide core of the RHIM and is likely to disrupt RHIM-mediated protein interaction and signaling.^5^ In addition, missense mutations in the kinase domain of RIPK1 that might alter its signaling function have also been found in different types of cancers. Moreover, Murphy *et al.*^42^ show that two MLKL mutations found in human cancers, F398I and L291P, represent non-functional and loss-of-function mutants for necroptosis, respectively. Hence, inhibition of RIPK and MLKL-dependent necroptosis may be advantageous for evasion of immune surveillance and cancer growth. In agreement with this hypothesis, reduced expression of MLKL is associated with poor survival in patients with pancreatic adenocarcinoma.^43^ It is noteworthy that although RIPK1 expression is reduced in colon cancer, its expression is elevated in lung cancer and glioblastoma tissues.^44, 45^ RIPK1 is an enigmatic signal adaptor that can function as a death promoter, a survival factor, and has important roles in promoting inflammatory cytokine production.^46^ The divergent expression pattern of RIPK1 in different types of cancers is likely a consequence of its pleiotropic functions in multiple signaling pathways.

A previous study suggests that *Ripk3* promoter hypermethylation underlies the loss of *Ripk3* expression in human small cell lung cancer.^24^ In contrast, we found that demethylating agent and histone deacetylation inhibitor did not enhance RIPK1 and RIPK3 expression in all of the cancer cell lines tested. Interestingly, transcription of *Ripk1* and *Ripk3* was controlled by hypoxia, suggesting that hypoxia may be one of the mechanisms that regulate *Ripk1* and *Ripk3* expression in human colon cancer tissues. The reduced RIPK3 expression in cancers contrasts that in inflammatory diseases, which often exhibit increased RIPK3 expression.^47, 48^ This suggests that the primary effect of reduced RIPK1 and RIPK3 expression in colon cancers may be to minimize anti-cancer immune activation.

Several recent studies show that necroptosis may have a role in cancer cell response to chemotherapeutic agents.^49, 50, 51, 52^ For instance, Obatoclax, a small-molecule inhibitor of the anti-apoptotic Bcl-2 proteins, was reported to trigger autophagy-dependent necroptosis in acute lymphoblastic leukemia cells and rhabdomyosarcoma cells.^53, 54^ These studies suggest that triggering necroptosis is a viable alternative in cancer treatment.^55^ However, it is noteworthy that the conclusion that necroptosis is responsible for cell death observed in some of the studies is mainly drawn using the RIPK1 inhibitor Nec-1.^55, 56, 57, 58^ As RIPK1 kinase activity is involved in other signaling pathways and off-target effects of Nec-1 have been reported,^37, 59, 60^ the veracity of these studies requires further scrutiny. Indeed, we found that RIPK1 and RIPK3 are largely dispensable for colon cancer cell death induced by many common chemotherapeutic agents. Further support for this conclusion comes from the observation that RIPK1 and RIPK3 expression in the NCI-60 human cancer cell panel does not correlate with cellular response to anticancer drugs (Supplementary Table S2).^61^ Hence, current chemotherapeutic agents appear to preferentially activate other cell death pathways and are ineffective inducers of necroptosis.

Unlike caspases and the RIP kinases, cell death induced by doxorubicin or etoposide is partially dependent on cysteine cathepsins. Our results are consistent with reports that chemotherapeutic agent-induced DNA damage leads to cathepsin-dependent cell death.^62, 63, 64^ Cathepsins are lysosomal proteases that are released on lysosomal membrane permeabilization and are known inducers of regulated necrosis.^65^ However, not all chemotherapeutic agents invoke cathepsin-dependent cell death, highlighting the distinct mechanisms employed by different chemotherapeutic agents to elicit cell death. As such, combinatorial therapy with other cytotoxic agents, such as that with tumor necrosis factor-related apoptosis-inducing ligand,^52^ may be necessary for necroptosis-targeted therapies. Alternatively, pathway-specific drugs that enhance RIPK3 expression and more potently induce necroptotic cell death will be needed to fully harness the power of necroptosis in anti-cancer therapy.

## Materials and Methods

### Cells

Colon cancer (HT29, Colo205, HCT116, and SW480), breast cancer (MDA-MB-231, MDA-MB-435, and MCF7), cervical cancer (HeLS-S3), liver cancer (HepG2), and lung cancer (A549) cell lines were cultured in DMEM. T-cell leukemia (Jurkat and H9), histiocytic lymphoma (U937), chronic myelogenous leukemia (K562), acute myelogenous leukemia (KG-1), and B-cell lymphoma (BJAB and SKW6.4) cell lines were cultured in RPMI1640. Ten percent FCS, 2 mM glutamine, 100 units/ml penicillin, and 100 *μ*g/ml streptomycin were added to the media. For hypoxia experiments, cells were cultured under 1% O_2_ in a Ruskinn Hypoxia Chamber. To generate RIPK1 or RIPK3-knockdown cells, lentivirus was produced by transfecting 293T cells with pGIPZ vector carrying shRNA against RIPK1 (Open Biosystems, Lafayette, CO, USA, V2HS_17422) or RIPK3 (Open Biosystems, V2HS_77679) in combination with pMD2.G and psPAX2. pGIPZ vector carrying non-silencing shRNA was used as a control (Open Biosystems, RHS4346). Retrovirus carrying RIPK3 expression vector was produced by transfecting RIPK3/pBabe-puro vector with VSV-G and Gag/Pol vectors. Cells were transduced with the lentivirus or the retrovirus and subsequently selected by puromycin. HCT116 cells stably expressing RIPK3 were generated by transfecting with pEGFP-N1 carrying RIPK3 or mock vector and selecting with G418.

### Reagents

5AzadC, 5-FU, oxaliplatin, irinotecan, doxorubicin, and etoposide were obtained from Sigma (St. Louis, MO, USA). zVAD, Nec-1, DPQ, and PD150606 were obtained from Enzo Life Sciences (Farmingdale, NY, USA). LBW242 and BV6 are kind gifts from Novartis (Cambridge, MA, USA) and Genentech (San Francisco, CA, USA), respectively.

### Colon cancer tissue specimens

Nine pairs of frozen tumor and adjacent normal tissues from patients diagnosed clinically with colon cancer were obtained from the UMSS cancer center tissue bank. Isolated tissues after surgical operation were stored in RNAlater for RNA extraction. Paraffin-embedded tissue from patient 2 was also obtained from the UMSS cancer center tissue bank. Clinical background of each patient was summarized in Supplementary Table S1.

### Quantitative PCR

Total RNA from tissues and cells were extracted using RNeasy kit (Qiagen, Valencia, CA, USA). cDNA was synthesized using Superscript III (Invitrogen, Carlsbad, CA, USA). Real-time PCR analysis using iQ SYBR Green supermix (Bio-Rad Laboratories, Hercules, CA, USA) was performed on C1000 thermal cycler and CFX96 real-time system (Bio-Rad Laboratories). The following primers were used: F 5′-GGCATTGAAGAAAAATTTAGGC-3′, R 5′-TCACAACTGCATTTTCGTTTG-3′ for *Ripk1*; F 5′-GACTCCCGGCTTAGAAGGACT-3′, R 5′-CTGCTCTTGAGCTGAGACAGG-3′ for *Ripk3*; F 5′-AGAGCTCCAGTGGCCATAAA-3′, R 5′-TACGCAGGATGTTGGGAGAT-3′ for *Mlkl*; F 5′-TTTGATGGAGTGCAGCTTTG-3′, R 5′-CTCCTTTCCTGCGTCACACT-3′ for *Cyld*; F 5′-AAGTGCCCAAACTTCACAGC-3′, R 5′-TACTGTGCAGTCATCGTGGG-3′ for *Casp8*; F 5′-GAGCTGCTCGCCTCCCT-3′, R 5′-TCTCCAATCTTTCCCCACAT-3′ for *Fadd*; F 5′-GAAATCCCATCACCATCTTCCAGG-3′, R 5′-GAGCCCCAGCCTTCTCCATG-3′ for *Gapdh*.

### Immunohistochemistry

Immunohistochemical staining was performed on formalin-fixed paraffin-embedded tissues using anti-RIPK1 (Santa Cruz, Dallas, TX, USA) and RIPK3 antibodies in the UMMS Morphology Core facility. Anti-RIPK3 antibody was generated against C-terminal peptide of human RIPK3. Nuclei were conterstained with hematoxylin.

### Western blotting

Whole-cell extracts were prepared from cells using RIPA lysis buffer. Western blotting analysis was performed using anti-RIPK1 (BD Biosciences, San Jose, CA, USA), RIPK3, p21 (Santa Cruz), cIAP1/2 (R&D Systems, Minneapolis, MN, USA), PARP-1 (BD Biosciences) and cleaved PARP-1 (Cell Signaling Technology, Danvers, MA, USA) antibodies. Anti-HSP90 (BD Biosciences) and *β*-actin (Prosci, Poway, CA, USA) antibodies were used as a loading control.

### Cell death assay

Cell death assay was performed by CellTiter-Glo Luminescent Cell Viability Assay (Promega, Madison, WI, USA), CellTiter96 Aqueous Non-Radioactive Cell Proliferation Assay (Promega), or FACS using propidium iodide and Annexin V-FITC. Cells were pretreated with 10 *μ*M zVAD, 3 *μ*M Nec-1, or 3 *μ*M GSK'840 for 1 h before treatment with chemotherapeutic agents or necroptosis induction, unless otherwise stated. Cells were treated with 100 *μ*M 5-FU, 50 *μ*M oxaliplatin, 50 *μ*M irinotecan, 5 *μ*M doxorubicin, or 50 *μ*M etoposide for 48 h, unless otherwise stated. MDA-MB-231 cells were treated with 100 *μ*M etoposide for 48 h. Necroptosis was induced by pretreatment with 10 *μ*M zVAD and 2.5 *μ*M LBW242 for 1 h followed by treatment with TNF overnight, unless otherwise stated.

### Caspase activity assay

Caspase activity was determined by caspase 3 colorimetric protease assay kit (MBL, Woburn, MA, USA), according to the manufacturer's instructions.

### Statistical analysis

*P-*values were calculated by Wilcoxon matched-pairs signed-rank test or unpaired *t*-test with Welch's correction. Spearman correlation coefficients were used to evaluate relations between mRNA and protein expressions. *P-*values <0.05 were considered statistically significant.

## Abbreviations

RIPK: receptor interacting protein kinase
Smac: second mitochondria-derived activator of caspase
shRNA: short hairpin RNA
5AzadC: 5-aza-2′-deoxycytidine
TSA: trichostatin A
NF-*κ*B: nuclear factor-*κ*B
PARP-1: poly (ADP-ribose) polymerase-1
