# Supplementary material for: Differential roles of RIPK1 and RIPK3 in TNF-induced necroptosis and chemotherapeutic agent-induced cell death
Source: Cell Death Dis. 2015 Feb 12;6(2):e1636–. doi: 10.1038/cddis.2015.16 (PMC4669795; doi:10.1038/cddis.2015.16)
Supplement: Supplementary Information [file cddis201516x1.pdf]

Supplementary Fig. S1

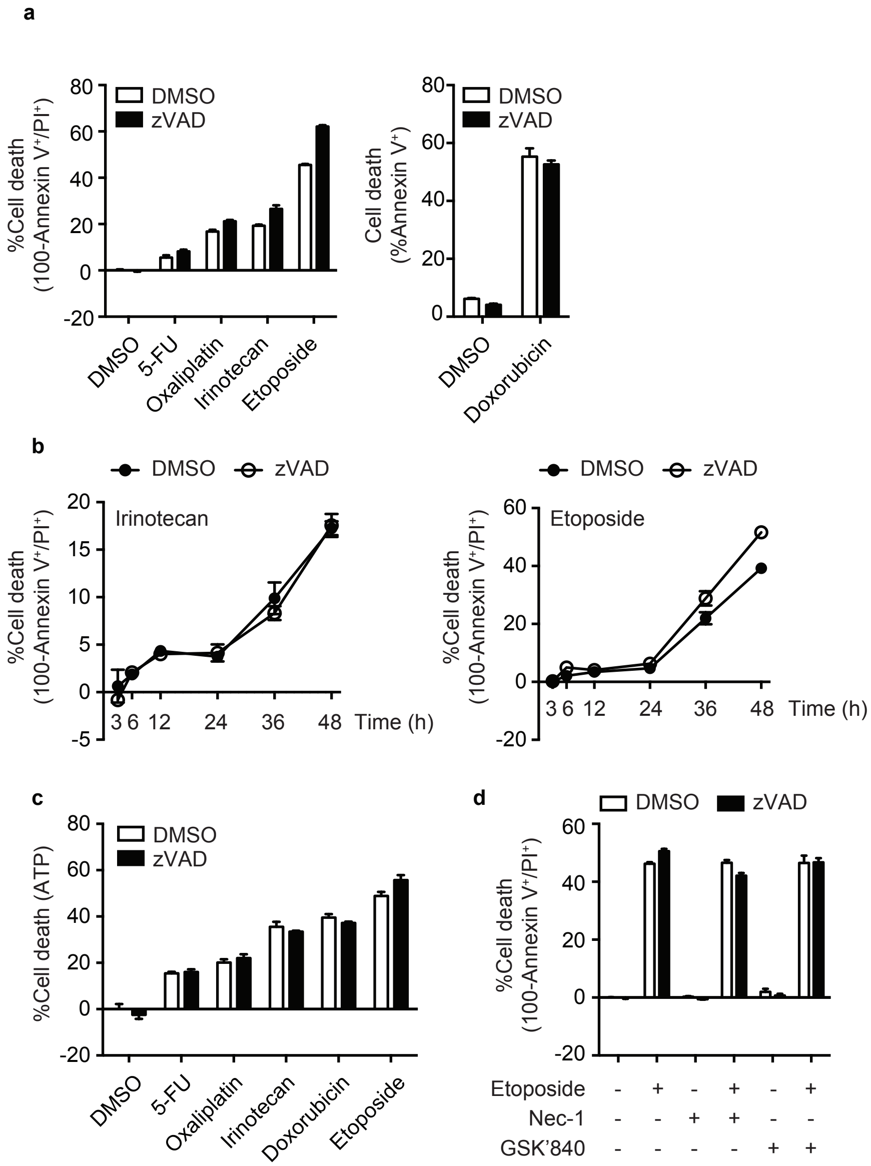

Supplementary Figure S1. The caspase inhibitor, z-VAD-fmk, does not block chemotherapeutic agent-induced cell death.

HT29 cells were pretreated with (**a, b, d**) 10 μM or (**c**) 100 μM z-VAD-fmk (zVAD) before treatment with the indicated chemotherapeutic agents for (**a, c, d**) 48 hours or (**b**) the indicated time. (**d**) Cells were pretreated with zVAD and either 3 μM Nec-1 or 3 μM GSK'840 as indicated. Cell death was determined by (**c**) CellTiter-Glo Luminescent Cell Viability Assay or (**a, b, d**) FACS using Annexin V-FITC and/or PI. Error bars indicate SEM (n=3).

Supplementary Fig. S2

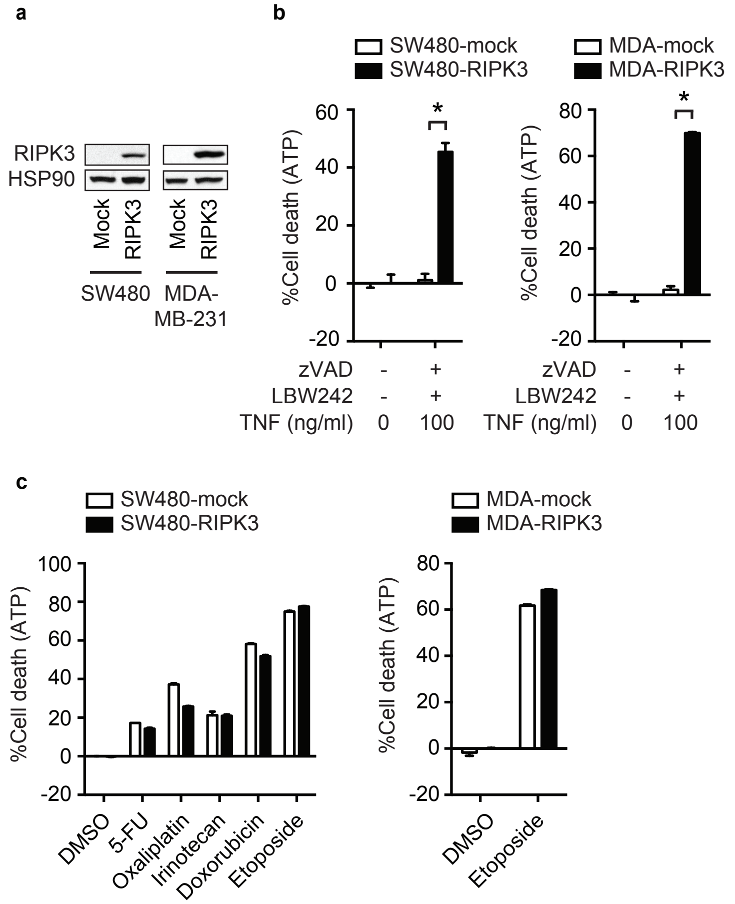

Supplementary Figure S2. Overexpression of RIPK3 does not confer protection against chemotherapeutic agent-induced cell death.

SW480 colon cancer cells and MDA-MB-231 breast cancer cells were transduced with retrovirus encoding RIPK3. (**a**) The expression of RIPK3 was confirmed by western blotting. (**b**) Cells were pretreated with 10 μM z-VAD-fmk (zVAD) and 2.5 μM LBW242 for 1 hour before addition of TNF for 14 hours. (**c**) Cells were treated with the indicated chemotherapeutic agents for 48 hours. Cell death was determined by CellTiter-Glo Luminescent Cell Viability Assay. Error bars indicate SEM (n=3). Asterisks: $p<0.05$.

Supplementary table S1. Clinical backgrounds of patients enrolled in this study.

	Anatomic location	Histologic type	Histologic grade	Pathologic classification			size (cm)			Relative expression in tumor tissues[1]	
										Ripk1	*Ripk3*
1	Cecum	Adenocarcinoma	G3 poorly differentiated	T3	N0	Mx	6	5.5	0.6	0.387	0.296
2	Ascending colon	Mucinouse carcinoma with signet ring cells	G3-G4 poorly differentiated	T3	N0	Mx	10.5	9.8	3	0.246	0.152
3	Ascending colon	Adenocarcinoma	G3 poorly differentiated	T3	N1	Mx	7.5	4.2	1.2	0.944	0.562
4	Ascending colon	Adenocarcinoma	G3 poorly differentiated	T3	N2	Mx	12.3	6.2	2	0.279	0.372
5	Ascending colon	Medullary carcinoma	G3 poorly differentiated	T3	N0	Mx	6.5	5.5	1	0.393	0.264
6	Transverse colon	Adenocarcinoma	G2 moderately differentiated	T3	N0	Mx	6	5.8	1	0.540	0.373
7	Sigmoid colon	Adenocarcinoma	G2 moderately differentiated	T3	N1	Mx	5.8	5.5	1.5	0.714	0.352
8	Cecum	Adenocarcinoma	G2 moderately differentiated	T3	N1	Mx	8.5	5.2	1.5	0.718	0.482
9	Sigmoid colon	Adenocarcinoma	G2 moderately differentiated	T4b	N0	Mx	5.7	5.3	1.2	0.686	0.612

[1]The expression level in the paired nomal colon tissues was defined as 1.

Supplementary table S2. Correlation between drug sensitivity and expression level of RIPK1 and RIPK3

	Ripk1	*Ripk3*
5-FU	-0.079	0.172
Oxaliplatin	-0.097	0.11
Irinotecan	-0.087	-0.09
Doxorubicin	0	-0.014
Etoposide	-0.069	-0.064

The data were extracted from CellMiner website (http://discover.nci.nih.gov/cellminer/home.do).
r value from Pearson's correlation coefficient is shown.
r value more than 0.334 is considered statistically significant.
